# Gap Junction Intercellular Communication Negatively Regulates Cadmium-Induced Autophagy and Inhibition of Autophagic Flux in Buffalo Rat Liver 3A Cells

**DOI:** 10.3389/fphar.2020.596046

**Published:** 2020-11-26

**Authors:** Hui Zou, Junzhao Yuan, Yi Zhang, Tao Wang, Yan Chen, Yan Yuan, Jianchun Bian, Zongping Liu

**Affiliations:** ^1^ College of Veterinary Medicine, Yangzhou University, Yangzhou, China; ^2^ Jiangsu Co-innovation Center for Prevention and Control of Important Animal Infectious Diseases and Zoonoses, Yangzhou, China; ^3^ Joint International Research Laboratory of Agriculture and Agri-Product Safety, The Ministry of Education of China, Yangzhou University, Yangzhou, China

**Keywords:** cadmium, autophagy, autophagic flux, gap junction intercellular communication, buffalo rat liver 3A cells

## Abstract

Cadmium is an important environmental pollutant that poses a serious threat to the health of humans and animals. A large number of studies have shown that the liver is one of the important target organs of cadmium. Stimulation of cells can lead to rapid changes in gap junction intercellular communication (GJIC) and autophagy. Previous studies have shown that cadmium can inhibit GJIC and induce autophagy. In order to understand the dynamic changes of GJIC and autophagy in the process of cadmium-induced hepatotoxic injury and the effects of GJIC on autophagy, a time-gradient model of cadmium cytotoxicity was established. The results showed that within 24 h of cadmium exposure, 5 μmol/L cadmium inhibited GJIC by down regulating the expression levels of connexin 43 (Cx43) and disturbing the localization of Cx43 in Buffalo rat liver 3A (BRL 3A) cells. In addition, cadmium induced autophagy and then inhibited autophagic flux in the later stage. During this process, inhibiting of GJIC could exacerbate the cytotoxic damage of cadmium and induce autophagy, but further blocked autophagic flux, promoting GJIC in order to obtain the opposite results.

## Introduction

Cadmium (Cd) is a significant pollutant caused by the improper emissions in the industry. It is difficult for animals and humans to excrete Cd as it can be present in the body for as long as 30 years (Nordberg, 2009). Previous studies have shown that the liver and kidney are the two main target organs of Cd toxicity and that the degree of damage to the liver is far higher than that of the kidney, and cadmium can even cause liver cancer (Song et al., 2016; Liu et al., 2017a; Wang et al., 2017; Wang et al., 2018; Gong et al., 2019; Wang et al., 2019). The amount that Cd stored in the liver is approximately one third of the total amount of the whole body (Marettova et al., 2012).

The role of the liver in animal metabolism and energy distribution is crucial, and the function of hepatocytes is greatly dependent on autophagy. It has been shown that hepatocytes maintain at a basic level of autophagy and that the fluctuation of autophagy levels can largely affect their physiological functions. The changes in the function of autophagy are also closely associated with the hepatocyte disease (Czaja et al., 2013). Autophagy plays a compensatory role in acute liver injury and regulates chronic liver diseases including alcoholic (ALD) and nonalcoholic fatty liver diseases (NAFLD) (Gual et al., 2017; Shi et al., 2017). During the pathogenesis of hepatitis B and C virus (HBV and HCV), the virus completes its life cycle by regulating the levels of autophagy in hepatocytes (Tang et al., 2009). Meng *et al.* demonstrated that when human embryonic liver cell lines (WRL-68) were treated with a Cd concentration gradient of 0–10 μmol/L *in vitro*, the autophagic protein marker LC3II indicated a gradual increase within 12 h (Meng et al., 2015). Zhang *et al.* reported that following 24 h treatment of chicken liver cells with 5 μmol/L of Cd, the number of MDC-labeled autophagic vesicles was significantly increased as demonstrated by fluorescence microscopy (Zhang et al., 2017). Our previous study demonstrated that treatment of Buffalo rat liver 3A (BRL 3A) cells for 6 h with 5 μmol/L Cd induced an increase in the autophagy levels (Zou et al., 2015c). Moreover, Cd obstructed the autophagic flux by inhibiting the fusion of autophagosomes with lysosomes, which exacerbated the Cd-induced cytotoxicity in alpha mouse liver 12 (AML12) cells (Zou et al., 2020).

The vigorous metabolic activity that takes place in liver cells determines that gap junction intercellular communication (GJIC) is another key mechanism to maintain homeostasis in liver tissues. GJIC constitutively allows the adjacent cell to deliver molecules directly, such as metabolites, nucleotides, nutrients, and secondary messengers that function in apoptosis, gene expression, inflammatory response and cellular growth (Decrock et al., 2009). Cx43 is sensitive to several environmental pollutants, biological toxins, organic solvents and pesticides, all of which can inhibit the expression of connexins and further downregulate GJIC function (Vinken et al., 2009).

Cd induces calcium overload in rat renal proximal tubule cells and the inhibition of GJIC function by Cd occurs prior to the induction of Cd-cytotoxicity. Therefore, the inhibition of GJIC by Cd is considered a marker of renal toxicity (Fukumoto et al., 2001). Jeong *et al.* reported that Cd treatment *in vivo* resulted in reduced GJIC function in mouse liver tissues in a concentration- and time-dependent manner (Jeong et al., 2000). We further found that Cd treatment increased the levels of Ca^2+^ in the cytoplasm, which is the main reason for the induction of hepatotoxicity and apoptosis in the BRL 3A cell line (Zou et al., 2015b).Therefore, it is an indisputable fact that Cd exposure causes decreased GJIC function and reduced connexins expression.

Our previous work demonstrated that treatment with Cd for 6 h could inhibit GJIC and reduce the expression levels of Cx43 in a dose-dependent manner in BRL 3A cells, whereas Cd-induced apoptosis in BRL 3A cells might be associated with inhibition of GJIC. We further showed that Cd triggered autophagy in a dose-dependent manner and that autophagy exerted a protective effect on the cytotoxicity of Cd in BRL 3A cells (Zou et al., 2015c). It is not clear whether a specific interaction between GJIC and autophagy exists or whether a direct regulatory effect is present between them. In view of the complexity of GJIC and cellular autophagy, the present study aimed to establish a time-gradient model of cadmium-induced liver cell damage *in vitro*, in order to further examine the intrinsic association between GJIC and autophagy.

## Material and Methods

### Materials

Cd acetate hydrate (Cd, 229490), Lucifer Yellow CH dilithium salt (LY, L0259), Rhodamine B isothiocyanate-Dextran (RD, R9379), all-trans-retinoic acid (ATRA, PHR1187), 18-β-glycyrrhetinic acid (GA, G10105), anti-LC3B (L7543) and anti-p62/SQSTM1 (P0067) were obtained from Sigma-Aldrich (St. Louis, MO, United States); anti-β-actin (4970S), anti-Atg7 (2631S), Anti-Beclin-1 (3738S), anti-Cx43 (3512S), anti-Phospho-Cx43 (Ser368) (3511S) and anti-rabbit IgG (7074), were obtained from Cell Signaling Technology Inc. (Danvers, MA, United States); EGFP-pmCherry-LC3 plasmid was obtained from HedgehogBio (Shanghai, China). DMEM (Gibco, 12800–017), FBS (Gibco, 10437–028) and Lipofectamine 3000 (L3000-015) were obtained from Thermo Fisher Scientific (Waltham, MA, United States); Alexa Fluor 488-labeled goat anti-rabbit IgG (A0423) and Cy3-labeled goat anti-rat IgG (A0507) were obtained from Beyotime (Shanghai, China). The remaining materials were of analytical grade.

### Cell Culture

BRL 3A cells were obtained from the Cell Bank of the Institute of Biochemistry and Cell Biology (Shanghai, China). Cells were cultured in DMEM supplemented with 10% FBS and incubated at 37°C in a 5% CO_2_ atmosphere. BRL 3A cells were treated at 90% confluence in the subsequent experiments.

In the present study, BRL 3A cells were transfected with the EGFP-pmCherry-LC3 plasmid (500 ng/well) for 12 h. The cells were transfected using Lipofectamine 3000 transfection reagent and subsequently LC3 puncta (EGFP-LC3 represents autophagosomes and pmCherry-LC3 represents autolysosomes) were identified using the confocal microscope (TCS SP8 STED; Wetzlar, Hessen, GER).Following successful transfection, the cells were subjected to different treatments according to the experimental design.

### Cell Viability Assay

Real time analysis of cytotoxicity was determined by the xCELLigence system, which is a real-time cell analyzer (RTCA; Roche Applied Science, Basel, Switzerland) operated according to the manufacturer’s instructions (Xing et al., 2005). BRL 3A cells were treated with 5 μmol/L Cd as previously described (Zou et al., 2015c). The nuclei were stained with 5 mg/ml Hoechst33258 according to the manufacturer’s instructions (Beyotime, Jiangsu, China) and subsequently observed by fluorescent microscopy (Leica DMI 3000B, Solms, Germany).

### Transmission Electron Microscopy

Transmission electron microscopy (CM 100, Philips, Holland) was used in order to assess the ultrastructure of the cells. Following treatment, the cells were collected by trypsinization. The samples were embedded into the resin following a series of fixed treatment and subsequently the resin was embedded with the cells and sliced with a slicer. Following the double staining with uranium dioxide acetate and lead citrate, the resin was observed and photographed under a transmission electron microscope.

### Immunofluorescence Assay

BRL 3A cells were inoculated into 24-well plates with coverslips and following cellular fusion for approximately 90% of the cells, the original culture medium was replaced with serum-free medium. The treatments were conducted according to the experiment design. Following treatment, the cells were washed with 0.01 mol/L ice-cold PBS for three times and fixed in 4% paraformaldehyde solution, 0.4% Triton X-100 film and 5% BSA. These solutions were used to penetrate the cellular membranes and block the corresponding epitopes, respectively. The cells were incubated with diluted primary antibodies overnight at 4°C. Following washing with PBS, the secondary antibody labeled with FITC (1:20 dilution) was incubated with the cells at room temperature for 1 h in the dark. DAPI was added for 15 min in the last step and the film was sealed and imaged using confocal microscopy (TCS SP8 STED; Wetzlar, Hessen, GER).

### Western Blot Analysis

Following treatment, the cells were washed three times with ice-cold PBS and subsequently extracted by ultra sonicationin Radio Immunoprecipitation Assay (RIPA) lysis buffer. The total cellular protein concentration was determined by the bicinchoninic acid (BCA) protein assay kit (Beyotime, China). Equal amounts of total proteins (20–40 μg) were separated by SDS-PAGE and subsequently transferred to polyvinylidene difluoride (PVDF) membranes. The membranes were blocked with 5% non-fat milk in Tris-buffered saline with 0.05% Tween-20 (TBST) at room temperature for 2 h followed by incubation with the primary antibodies at 4°C overnight. The following morning, the membranes were incubated with the diluted secondary antibody for 2 h at room temperature. The protein bands were visualized using an enhanced chemiluminescence (ECL) detection kit and the Grayscale values of the protein bands were analyzed using the Image J software (National Institutes of Health, United States).

### Scrape Loading/Dye Transfer Assay

A scrape-loading/dye-transfer method (SL/DT) was used to determine GJIC following treatment, which was performed as originally described by El-Fouly et al. (1987). In brief, BRL3A cells were washed for three times by PBS and subsequently the cells were scraped, incubated with PBS containing LY (0.5 mg/ml) and RD (2.5 mg/ml) at 37°C for 3 min and fixed with 4% paraformaldehyde. Finally, the distance from the scraped edge to the neighboring cells was measured by fluorescence microscopy (Leica DMI3000 B, Solms, Germany). The data were expressed as the mean ± standard deviation (*n* = 6).

### Statistical Analysis

The data were analyzed by one-way analysis of variance (ANOVA) using the GraphPad Prism 6 software (GraphPad Software, United States) and presented as the mean ± SD. A *p* < 0.05 represented a significant difference and a *p* < 0.01 represented a highly significant difference.

## Results

### Cadmium Exposure Induces Cytotoxicity in Buffalo Rat Liver 3A Cells

**FIGURE 1:**
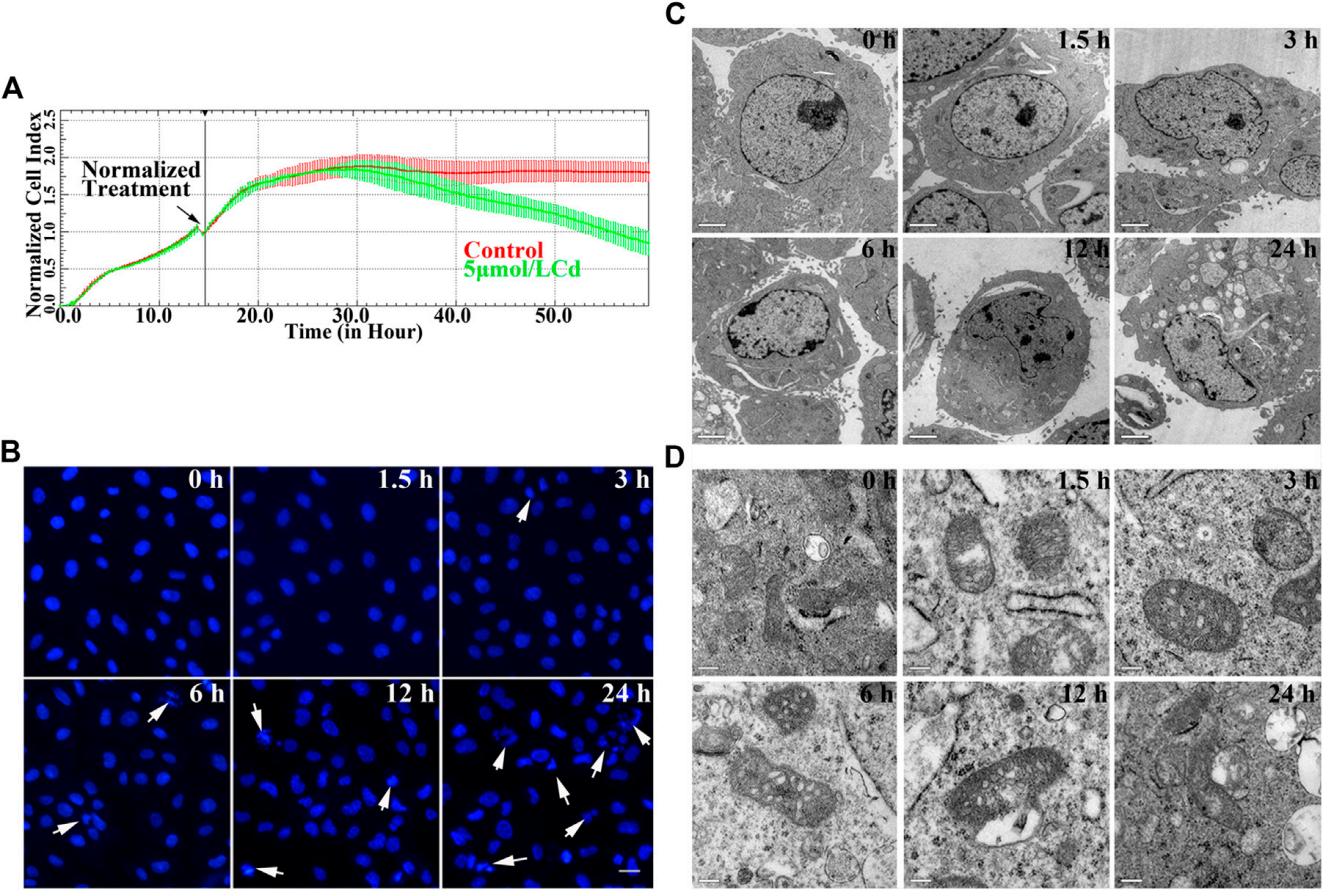
Cd exposure induces cytotoxicity in BRL 3A cells. **(A)** Effects of 5 μmol/L Cd on cell index in BRL 3A cells. The data were normalized at the time of Cd treatment. The error bars represents the standard deviation (*n* = 6). **(B)** BRL 3A cells were treated with5 μmol/l for different time periods and stained with Hoechst 33258. The white arrows indicate damaged nucleus. Scale bar = 20 μm. **(C,D)** Cd-induced ultrastructure changes at different time periods in BRL 3A cells. The images were captured by transmission electron microscopy. The scale bar in the nuclei was 2 μm, whereas the scale bar in the mitochondria was 0.2 μm.

Real-time cell analysis was conducted with the application of the xCELLigence system and the results are shown in Figures 1A. Exposure of the cell to 5 μmol/L Cd resulted in a decrease of cell index following 12 h in a time-dependent manner. The morphological changes of the BRL 3A cell nuclei by Cd were observed using both fluorescent nuclear staining and transmission electron microscopy. The cell nuclei of the treated group were deformed, shrunk, disintegrated with condensed chromatin following Cd treatment compared with the corresponding morphology of the cells in the control group (Figures 1B,C). The ultra-structural changes of the mitochondria showed that the swelling of the cristae and the deformation and vacuolization of the mitochondria were observed in the treated group. Increased Cd concentration, the cell damage was increased (Figures 1D).

### Cadmium Exposure Induces Autophagy and Then Blocks Autophagic Flux in Buffalo Rat Liver 3A Cells

**FIGURE 2:**
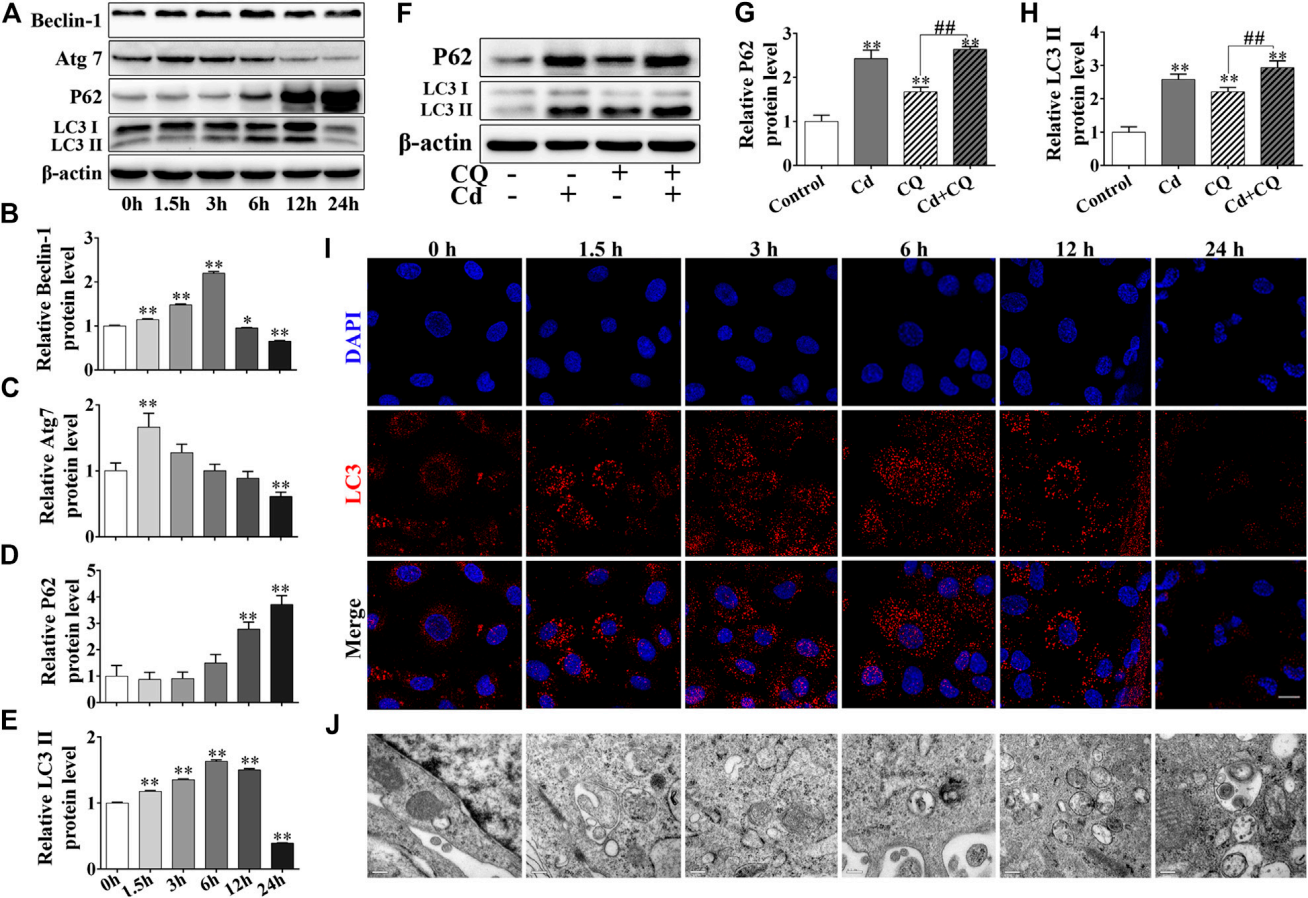
Cd induces autophagy and blocks autophagic flux in BRL 3A cells. **(A–E)** Cd treatment for different time periods affected the expression levels of autophagy-associated proteins in BRL 3A cells. *p < 0.05, **p < 0.01compared with the control group. **(B–E)** share the abscissa annotation. **(F–H)** The levels of LC3 and P62 following combination treatment of Cd and CQ were evaluated in BRL 3A cells. **p < 0.01compared with the control group, ^##^ *p* < 0.01 compared with the indicated group. **(I)** Following treatment with 5 μmol/L Cd, LC3 puncta (red) were observed and photographed under the fluorescence microscope. Scale bar = 20 μm. **(J)** Following treatment with Cd, the changes in the autophagosomes in BRL 3A cells were observed and imaged under the transmission electron microscope. Scale bar = 0.2 μm. **(I,J)** share the abscissa annotation.

BRL 3A cells were treated with 5 μmol/L Cd for various time durations (0, 1.5, 3, 6, 12, and 24 h). The results of the western blot analysis indicated that the expression levels of autophagy-related proteins ATG7, Beclin-1 and LC3II increased first and then decreased. The relative expression levels of Beclin-1 and LC3II reached their highest levels following 6 h of Cd treatment, while Atg7 reached the highest levels following 1.5 h of Cd treatment. The relative expression levels of these three proteins were decreased and were significantly lower than those of the control group (*p* < 0.01, Figures 2A–E). The addition of chloroquine (CQ), which is known as an autophagic flux inhibitor, significantly enhanced the accumulation of P62 and further increased LC3II expression (*p* < 0.01, Figures 2F–H). Cd treatment increased the number of LC3 puncta in a time-dependent manner, whereas the amount of LC3 puncta reached its peak following 12 h of Cd treatment, as demonstrated by immunofluorescence staining. However, a low number of LC3 puncta were observed and the cell nuclei were shrunk and deformed following 12 h of Cd treatment (Figures 2I). Transmission electron microscopy revealed the accumulation of autophagosomes following increased exposure of the cells to Cd (Figures 2J).

### Cadmium Inhibits Gap Junction Intercellular Communication in Time Dependent Manner in Buffalo Rat Liver 3A Cells

**FIGURE 3:**
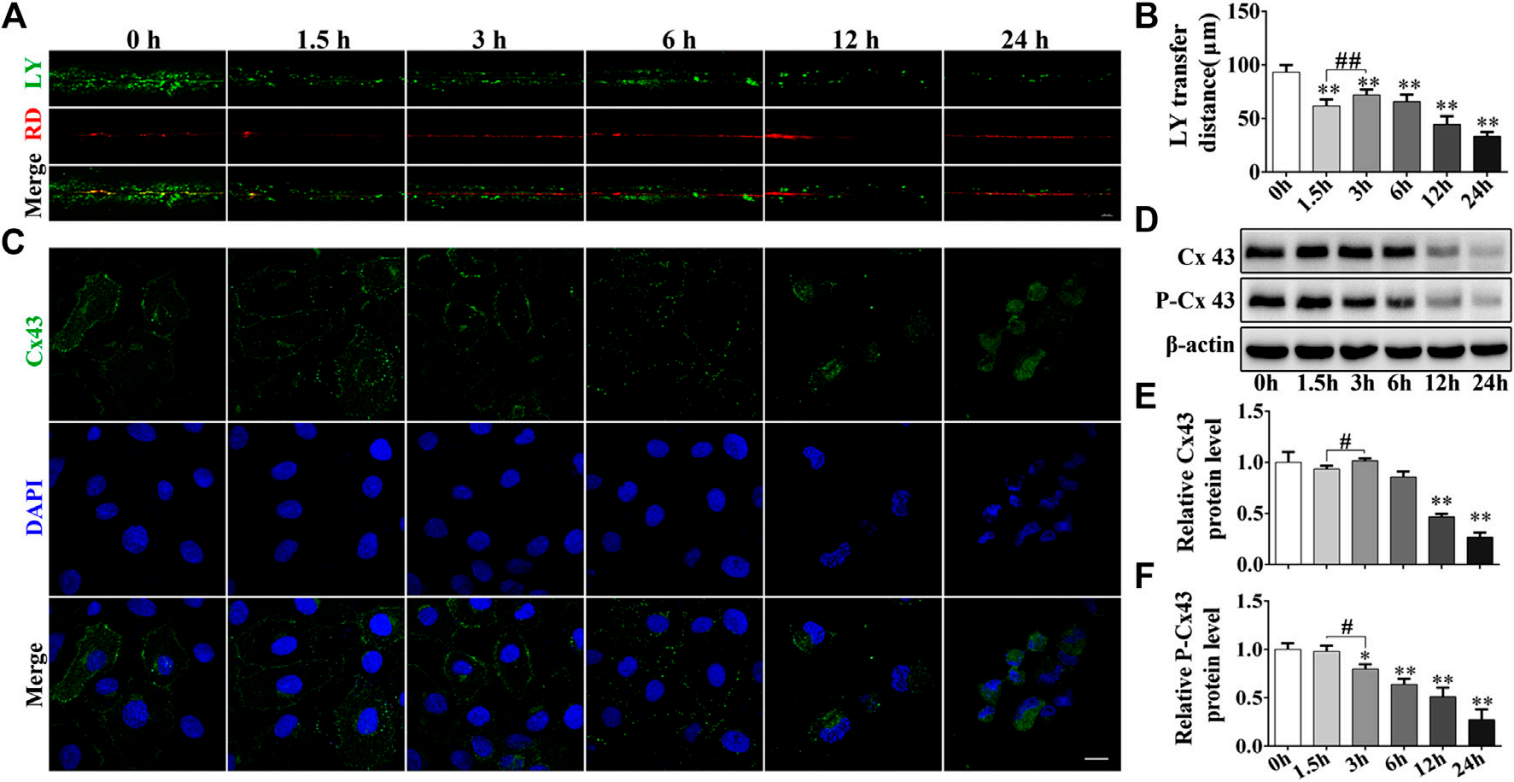
Cd inhibits the GJIC in BRL 3A cells. **(A)** Following treatment with 5 μmol/L Cd for different time periods, GJIC was measured by the SL/DT method (scale bar = 50 μm). **(B)** Quantification of data obtained for the LY transfer distance depicting the spread of the dye from each side of the wound area (*n* = 6). **(C)** 5 μmol/L Cd disrupts Cx43 distribution in BRL 3A cells.Cx43 expression (green) and the presence of the nuclei (blue) were observed and imaged using confocal microscopy. Scale bar = 10 μm. **(D–F)** Cd treatment at different time periods affects the levels of connexin expression in BRL 3A cells. Compared with the control group, *p < 0.05, **p < 0.01; compared with the indicated group, ^#^ *p* < 0.05, ^##^ *p* < 0.01.

By performing the scrape-loading dye transfer assay we deduced that Cd treatment exhibited an apparent inhibitory effect to GJIC in a time-dependent manner for a total exposure period of 24 h (*p* < 0.01). It is interesting to note that exposure of the cells to Cd for 3 h increased GJIC compared with that noted at 1.5 h (*p* < 0.01, Figures 3A,B). Immunofluorescence staining for Cx43 demonstrated that 5 μmol/L Cd treatment for different time periods could decrease the expression levels of Cx43. Following treatment of the cells with Cd for 6 h, the expression levels of Cx43 at the cell membrane exhibited a linear and uniform distribution, whereas following treatment of the cells with Cd for 12 h or 24 h, Cx43 concentrated around the nucleus compared with the corresponding expression noted in the normal cells (Figures 3C). Subsequently, the expression levels of Cx43 and P-Cx43 (Ser368) were detected at different time points after cadmium treatment. The expression levels of Cx43 were decreased significantly following Cd treatment for 12 and 24 h compared with those of the control group (*p* < 0.01). No significant change was noted prior to Cd treatment for 12 h, while the expression levels of Cx43 following Cd exposure for 3 h were significantly higher than those noted at 1.5 h of Cd treatment. The expression levels of P-Cx43 were decreased in a time-dependent manner (Figures 3D–F).

### Gap Junction Intercellular Communication Regulates the Cytotoxicity Induced by Cadmium in Buffalo Rat Liver 3A Cells

**FIGURE 4:**
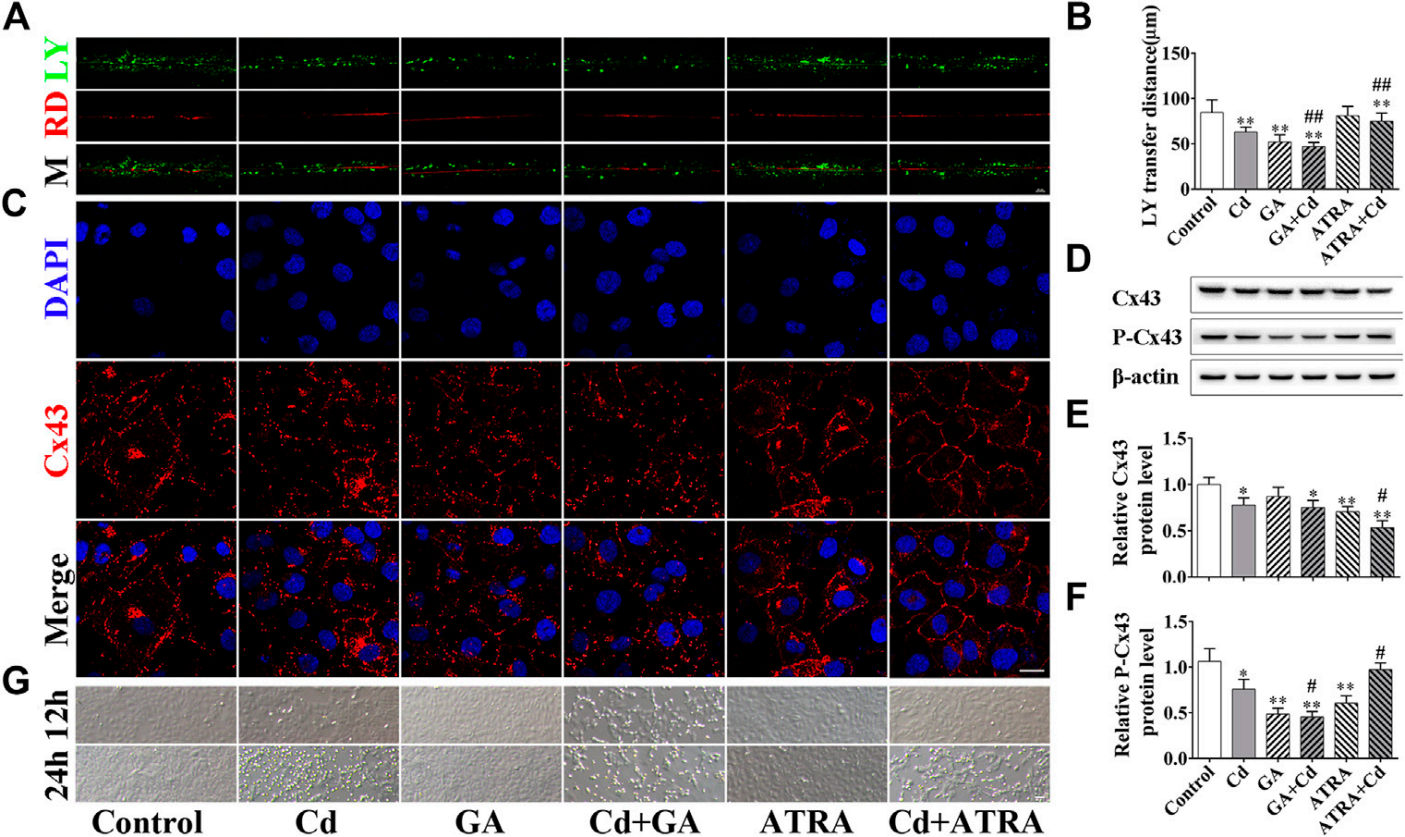
GJIC regulates the Cd-induced cytotoxicity in BRL 3A cells. Combination treatment of 5 μmol/L Cd with 5 μmol/l GA or 5 μmol/L ATRA for 6 h **(A,B)** GJIC was measured by the SL/DT method, Scale bar = 50 μm. **(C)** Immunofluorescence assay for the detection of Cx43 distribution. Cx43 (green) and nuclei (blue) were observed and imaged using confocal microscopy. (Scale bar = 20 μm). **(D–F)** The effects of GA and ATRA on Cx43 and p-Cx43 expression levels were analyzed by western blotting. **(G)** Bright field images indicating the effects of GA and ATRA on cell damage induced by Cd treatment. Scale bar = 50 μm. Compared with the control group, *p < 0.05, **p < 0.01; compared with the Cd treatment group, ^#^ *p* < 0.05, ^##^ *p* < 0.01.

To investigate the role of GJIC in Cd-induced cytotoxicity, GJIC was regulated by the inhibitor GA and the enhancer ATRA. The combined treatment of GA and Cd inhibited GJIC function, whereas the combined treatment of ATRA and Cd promoted the function of GJIC compared to the corresponding effects noted in the Cd group (Figures 4A,B, *p* < 0.01). The results of the immunofluorescence experiments are indicative of the GJIC function assay. The data indicated that GA increased the distribution of Cx43, whereas ATRA ameliorated it on the cell membrane (Figures 4C). Western blot analysis indicated that P-Cx43 (Ser368) levels were significantly decreased in the GA-Cd combined treatment group (*p* < 0.01), while they were significantly increased in the ATRA-Cd combined treatment group compared with the corresponding levels noted in the Cd single treatment group (*p* < 0.05). Cx43 levels were significantly decreased in the ATRA-Cd combined treatment group (*p* < 0.05) (Figures 4D–F). No cellular damage was evident with regard to the cell morphology at the 6 h Cd treatment period and the treatment time was extended to 12 and 24 h. The data at these time points indicated that GA further aggravated the cytotoxicity induced by Cd and that ATRA exhibited the opposite effects (Figures 4G).

### Gap Junction Intercellular Communication Negatively Regulates Cadmium-Induced Autophagy and Inhibition of Autophagic Flux in Buffalo Rat Liver 3A Cells

**FIGURE 5:**
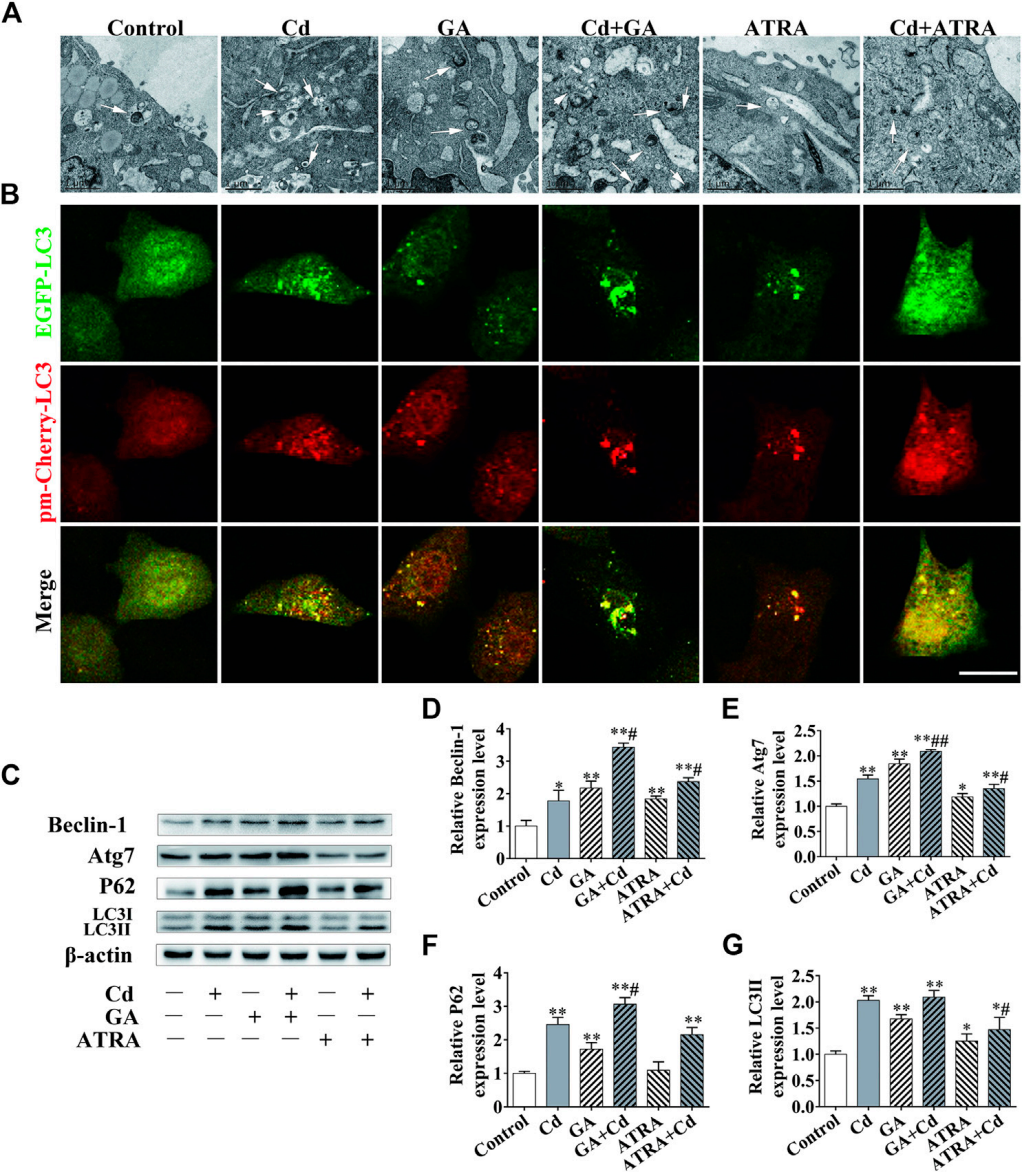
The association between GJIC and autophagy in cadmium induced hepatocyte injury. Combination treatment of 5 μmol/L Cd with 5 μmol/l GA or 5 μmol/L ATRA for 6 h, **(A)** Morphological observation of autophagosomes by transmission electron microscopy. Scale bar = 1 μm. **(B)** BRL 3A cells were transfected with the EGFP-pmCherry-LC3 plasmid and subsequently treated with Cd or an inhibitor/inducer of autophagy. LC3 puncta were observed by confocal microscopy Scale bar = 20 μm. **(C–G)** The effects of GA and ATRA on autophagy-associated protein expression levels were analyzed by western blotting. Compared with the control group, *p < 0.05, **p < 0.01; compared with the Cd treatment group, ^#^ *p* < 0.05, ^##^ *p* < 0.01.

The data indicated that the autophagic levels and those of Cx43 reached their highest value following Cd treatment for 6 h. As a result, the 6 h time period was selected as the main time point to study the association between autophagy and GJIC. To identify an interaction between GJIC and the autophagy levels in the process of Cd exposure, we evaluated the number and morphology of the autophagosomes in the cells following the application of GA and ATRA for 6 h. The combination of GA and Cd could apparently increase the number of autophagosomes, while the combination of ATRA and Cd exhibited the opposite effects (Figures 5A). LC3 was tracked by EGFP and pm-Cherry markers to monitor the autophagysome-lysosome fusion. The data demonstrated that combination of GA and Cd resulted in an increase in the LC3 orange puncta and in a patchy accumulation around the nucleus, while combination of ATRA and Cd resulted in a decrease in the LC3 puncta compared with the effects noted in the Cd treatment group (Figures 5B). Western blot analysis indicated that, compared with the Cd single treatment group, the combination of GA and Cd could significantly increase Beclin-1, Atg7 and P62 expression levels (*p* < 0.05, *p* < 0.01), whereas no changes were noted with regard to the LC3II expression levels, suggesting that inhibition of GJIC function could further block autophagic flux in Cd-induced cytotoxicity. Meanwhile, the combination of ATRA and Cd could significantly decrease Atg7 and LC3II expression levels compared with those of the Cd single treatment group (*p* < 0.05), and significantly increase Beclin-1 expression levels (*p* < 0.05), without affecting the expression levels of P62 (Figures 5D–G), suggesting that promotion of GJIC can inhibit Cd-induced autophagy.

## Discussion

According to the data of United States Geological Survey, more than 20,000 tons of heavy metal cadmium are produced in the world every year, which are mainly used in nickel-chromium batteries, paints, electroplating and alloys. Cd pollution in the environment can severely affect the health of animals and humans worldwide. Cd can induce ROS in hepatocytes, disrupt cellular oxidative homeostasis and subsequently lead to oxidative damage of hepatocytes. Furthermore, cadmium can induce apoptosis by activating multiple signaling pathways (Rikans and Yamano, 2000; Li and Xie, 2018). We chose BRL 3A cells for *in vitro* analysis in this study as BRL 3A cell line is one of the commonly used cell lines in hepatotoxicity researches. The dose of Cd used in this study (5 μmol/L) is based on previous studies (Zou et al., 2015c; Zhang et al., 2017). In the present study, 5 μmol/L of Cd were used to treat BRL 3A cells for 24 h and the effects of this heavy metal on the cells were detected at different time points. We found that Cd could reduce cell index, destroy cell ultrastructure and induce morphological changes in the apoptotic nuclei in a time-dependent manner (Figure 1).

Autophagy is a lysosome dependent degradation pathway, which can remove damaged organelles, misfolded proteins, pathogens and lipid droplets from cells and recycle them. Autophagy is considered a survival mechanism of the cells (Glick et al., 2010). When cells are in the state of starvation, hypoxia or toxic stimulation, pathogen invasion and other stress-inducing stimuli can activate autophagy in a rapid way. The substrates used for the process of autophagy can be cleared to provide energy and nutrients for the cells and this plays an important role in maintaining the integrity of organelles and proteins and the regulation of the immune system (Jin, 2006; Cuervo and Macian, 2012). The defects caused in the autophagosome can result in a dynamic imbalance leading to the progression of several neurodegenerative diseases, such as Alzheimer’s disease (AD). The major problem in these diseases is that metabolic waste accumulates in the cells and cannot be removed (Du et al., 2009; Cheng et al., 2015). Several reports have shown that Cd causes a significant increase in the levels of autophagy in the cells (Dong et al., 2009). Previous studies have confirmed that Cd can induce autophagy at a dose-response relationship in BRL 3A cells, the ability of cadmium to induce autophagy exerts a protective effect for the cells (Zou et al., 2015c). In the present study, the formation of autophagosomes was evident following treatment of the cells with Cd suggesting organelle degradation. Moreover, the expression levels of the autophagic proteins indicated a time-dependent increase within 24 h, which was consistent with previous studies. Following addition of chloroquine (CQ), the expression levels of P62 and LC3II were higher in the combined treatment group compared with those noted in the single Cd or CQ treatment groups, which indicated that cadmium not only promoted autophagy, but also blocked autophagic flux (Figure 2), similar results were obtained in the study of cadmium induced damage to AML12 cells (Zou et al., 2020).

GJIC is an important way of communication between mammalian cells. This process is essential for the hepatocytes in order to retain their cellular homeostasis. GJIC is regulated by a complex mechanism, which is not only related to the opening and closing of the GJ channels, but also to the location, expression and phosphorylation levels of connexin. Unlike normal membrane proteins, Cx43 turnover occurs at an extremely rapid rate and it is estimated that only 1.5–3 h are required for a full turnover cycle of Cx43. The maintenance of normal GJIC functionality requires continuous synthesis and degradation of Cx43.Therefore, several Cx43 inhibitors are designed to inhibit the synthesis of Cx43 in order to limit the GJIC function, such as GA and dynasore (Goubaeva et al., 2007; Girard et al., 2011; Vivian and Lau, 2016). Phosphorylation is an important chemical modification that is necessary for Cx43 function (Leithe et al., 2018). The phosphorylation of Cx43 at the ser368, 279 and 282 residues by PKC or ERK promotes the internalization of Cx43 (Solan and Lampe, 2018). Cx43 was a regulator of Cd toxicity and that it could decrease the levels of Akt phosphorylation at Thr308 and at Tyr326, which may contribute to the induction of cell apoptosis and to the inhibition of cell proliferation following chronic exposure of HK-2 cells to Cd. Moreover, Cx43 siRNA attenuated Cd-induced apoptosis and inhibited HK-2 cell proliferation (Ge et al., 2017). However, other study used low doses of Cd treatment and demonstrated that phosphorylation of Cx43 at ser368W was increased in human prostate epithelial cells (Luo et al., 2015; Liu et al., 2017b). These contradictory results may be associated with the different treatment doses of Cd and the different types of cells, which indicate the complex mechanism of action of the Cd-induced cytotoxicity. Our previous studies have shown that cadmium induces hepatocyte damage and causes damage to adjacent cells through GJIC (Zou et al., 2015a). In the present study, we found that Cd inhibited GJIC in 24 h, whereas a compensatory effect of GJIC was noted at 3 h (Figures 3B), which was accompanied with increased expression and cellular localization of Cx43 suggesting a recovery of the function of GJIC (Figures 3C–E). Subsequently, the data demonstrated that the addition of GA, an inhibitor of GJIC, could inhibit the opening of GJIC and disrupt the linear distribution of Cx43 on the cell membrane, significantly decreasing the levels of P-Cx43. The addition of the GJIC accelerator ATRA promoted the opening of GJIC and the linear distribution of Cx43 on the cell membrane, whereas it concomitantly reduced significantly the expression levels of Cx43 and increased the expression levels of P-Cx43, indicating that GJIC was not directly associated with the overall levels of Cx43 and that changes in the levels of P-cx43 were more sensitive to cadmium than those of Cx43. Furthermore, inhibiting of GJIC could exacerbate the toxic damage of cadmium, whereas promoting of GJIC could delay its cytotoxicity (Figure 4), suggesting that GJIC plays a dominant role in Cd-induced BRL 3A cell injury.

Apoptotic cells can transmit apoptotic signals through GJIC to induce apoptosis of adjacent cells (Contreras et al., 2004). However, whether cellular autophagy can induce further autophagy of adjacent cells through GJIC is not clear (Wang et al., 2015). Connexins can directly regulate the production of the autophagosome and its assembly (Merrifield and Kaksonen, 2014). P-Cx43 promotes autophagy in order to repair brain nerve cell damage in a rat model (Sun et al., 2015). Due to GJIC and autophagy being both changeable dynamic processes, the exact role of GJIC and autophagy in Cd-induced cell injury remains to be studied. Epifantseva and Shaw reported that the inhibition of mTOR could increase the levels of the GJA1-20k protein under stress conditions, leading to increased synthesis and transport of Cx43 to the cell membrane (Epifantseva and Shaw, 2018). Promoting autophagy can accelerate the degradation of Cx43 (Martins-Marques et al., 2014; Martins-Marques et al., 2015), and exacerbate Cd-induced GJIC inhibition (Zou et al., 2015c). In the present study, we found that the application of the GJIC inhibitor GA resulted in an increase of the amount of autophagosomes in BRL 3A cells, whereas the opposite phenomenon was noted with the usage of ATRA, which indicated a negative correlation between GJIC and autophagy. The results of the immunofluorescence analysis further indicated a significant negative correlation between autophagy and GJIC following cell exposure to Cd. Concomitantly, western blotting indicated that the GJIC inhibitor GA contributed to the accumulation of autophagic proteins (Figure 5).

In summary, the data indicated that Cd suppressed GJIC function in a time dependent manner, and promoted the levels of autophagy, then inhibited autophagic flux. Inhibiting GJIC exacerbates the cytotoxicity caused by Cd and further blocked the autophagic flux, promoting GJIC has the opposite effect. GJIC negatively regulates Cd-induced autophagy and inhibition of autophagic flux in BRL 3A cells.

## Data Availability Statement

The raw data supporting the conclusions of this article will be made available by the authors, without undue reservation.

## Author Contributions

Conception and design: HZ and ZL. Study conduct: HZ, JY, and YZ. Data collection: HZ and JY Data analysis: HZ, YZ, TW, and YC. Data interpretation: YY, JB, and ZL. Drafting manuscript: HZ. Revising manuscript content: HZ, YY, and JB. Approving final version of manuscript: ZL.

## Funding

This work was supported by the National Natural Science Foundation of China [Nos. 31702305, 31872533, 31772808, 31802260, and 31672620], the Natural Science Foundation of Jiangsu Province [No. BK20160458], the China Postdoctoral Science Foundation [2016M601900], a project funded by the Priority Academic Program Development of Jiangsu Higher Education Institutions (PAPD).

## Conflict of Interest

The authors declare that the research was conducted in the absence of any commercial or financial relationships that could be construed as a potential conflict of interest.

## References

[B1] Cheng X. T. Zhou B. Lin M. Y. Cai Q. Sheng Z.-H. (2015). Axonal autophagosomes recruit dynein for retrograde transport through fusion with late endosomes. J. Cell Biol. 209 (3), 377–386. 10.1083/jcb.201412046 25940348 PMC4427784

[B2] Contreras J. E. Sánchez H. A. Véliz L. P. Bukauskas F. F. Bennett M. V. L. Sáez J. C. (2004). Role of connexin-based gap junction channels and hemichannels in ischemia-induced cell death in nervous tissue. *Brain research* . Brain Res. Rev. 47 (1-3), 290–303. 10.1016/j.brainresrev.2004.08.002 15572178 PMC3651737

[B3] Cuervo A. M. Macian F. (2012). Autophagy, nutrition and immunology. Mol. Aspect. Med. 33 (1), 2–13. 10.1016/j.mam.2011.09.001 PMC3996457 21982744

[B4] Czaja M. J. Ding W. X. Donohue T. M. Jr. Friedman S. L. Kim J. S. Komatsu M. (2013). Functions of autophagy in normal and diseased liver. Autophagy 9 (8), 1131–1158. 10.4161/auto.25063 23774882 PMC3748187

[B5] Decrock E. Vuyst E. D. Vinken M. Moorhem M. V. Vranckx K. Wang N. (2009). Connexin 43 hemichannels contribute to the propagation of apoptotic cell death in a rat C6 glioma cell model. Cell Death Differ. 16, 151–163. 10.1038/cdd.2008.138 18820645

[B6] Dong Z. Li W. Xu J. Li Y. Yun Z. Zhang S. (2009). Promotion of autophagy and inhibition of apoptosis by low concentrations of cadmium in vascular endothelial cells. Toxicol in Vitro 23 (1), 105–110. 10.1016/j.tiv.2008.11.003 19061949

[B7] Du Y. Wooten M. C. Wooten M. W. (2009). Oxidative damage to the promoter region of SQSTM1/p62 is common to neurodegenerative disease. Neurobiol. Dis. 35 (2), 302–310. 10.1016/j.nbd.2009.05.015 19481605 PMC2718328

[B8] El-Fouly M. H. Trosko J. E. Chang C. C. (1987). Scrape-loading and dye transfer: a rapid and simple technique to study gap junctional intercellular communication. Exp. Cell Res. 168 (2), 422–430. 10.1016/0014-4827(87)90014-0 2433137

[B9] Epifantseva I. Shaw R. M. (2018). Intracellular trafficking pathways of Cx43 gap junction channels. Biochim. Biophys. Acta Biomembr. 1860 (1), 40–47. 10.1016/j.bbamem.2017.05.018 28576298 PMC5731482

[B10] Fukumoto M. Kujiraoka T. Hara M. Shibasaki T. Hosoya T. Yoshida M. (2001). Effect of cadmium on gap junctional intercellular communication in primary cultures of rat renal proximal tubular cells. Life Sci. 69 (3), 247–254. 10.1016/S0024-3205(01)01063-3 11441915

[B11] Ge Z. Diao H. Yu M. Ji X. Liu Q. Chang X. (2017). Connexin 43 mediates changes in protein phosphorylation in HK-2 cells during chronic cadmium exposure. Environ. Toxicol. Pharmacol. 53, 184–190. 10.1016/j.etap.2017.06.003 28651161

[B12] Girard E. Paul J. L. Beaune P. Lamaze C. Vedie B. (2011). Dynasore, a dynamin gtpase inhibitor, blocks late endosomal/lysosomal cholesterol trafficking in human macrophages. Atherosclerosis Suppl. 12 (1), 31–32. 10.1016/S1567-5688(11)70138-6

[B13] Glick D. Barth S. Macleod K. F. (2010). Autophagy: cellular and molecular mechanisms. J. Pathol. 221 (1), 3–12. 10.1002/path.2697 20225336 PMC2990190

[B14] Gong Z. G. Wang X. Y. Wang J. H. Fan R. F. Wang L. (2019). Trehalose prevents cadmium-induced hepatotoxicity by blocking Nrf2 pathway, restoring autophagy and inhibiting apoptosis. J. Inorg. Biochem. 192, 62–71. 10.1016/j.jinorgbio.2018.12.008 30599315

[B15] Goubaeva F. Mikami M. Giardina S. Ding B. Abe J. Yang J. (2007). Cardiac mitochondrial connexin 43 regulates apoptosis. Biochem. Biophs. Res. Commun. 352 (1), 97–103. 10.1016/j.bbrc.2006.10.177 PMC1829482 17107662

[B16] Gual P. Gilgenkrantz H. Lotersztajn S. (2017). Autophagy in chronic liver diseases: the two faces of Janus. Am. J. Physiol. Cell Physiol. 312 (3), C263–C273. 10.1152/ajpcell.00295.2016 27903585

[B17] Jeong S. H. Habeebu S. S. Klaassen C. D. (2000). Cadmium decreases gap junctional intercellular communication in mouse liver. Toxicol. Sci. 57 (1), 156–166. 10.1093/toxsci/57.1.156 10966522

[B18] Jin S. (2006). Autophagy, mitochondrial quality control, and oncogenesis. Autophagy 2 (2), 80–84. 10.4161/auto.2.2.2460 16874075

[B19] Leithe E. Mesnil M. Aasen T. (2018). The connexin 43 C-terminus: a tail of many tales. Biochim. Biophys. Acta 1860 (1), 48–64. 10.1016/j.bbamem.2017.05.008 28526583

[B20] Li J. Xie X. (2018). Inconsistent responses of liver mitochondria metabolism and standard metabolism in Silurus meridionalis when exposed to waterborne cadmium. Comp. Biochem. Physiol. C Toxicol. Pharmacol. 214, 17–22. 10.1016/j.cbpc.2018.08.003 30149079

[B21] Liu F. Wang X. Y. Zhou X. P. Liu Z. P. Song X. B. Wang Z. Y. (2017a). Cadmium disrupts autophagic flux by inhibiting cytosolic Ca^2+^-dependent autophagosome-lysosome fusion in primary rat proximal tubular cells. Toxicology 383, 13–23. 10.1016/j.tox.2017.03.016 28347754

[B22] Liu Q. Ji X. Ge Z. Diao H. Chang X. Wang L. (2017b). Role of connexin 43 in cadmium-induced proliferation of human prostate epithelial cells. J. Appl. Toxicol. 37 (8), 933–942. 10.1002/jat.3441 28176351

[B23] Luo C. Yuan D. Yao W. Cai J. Zhou S. Zhang Y. (2015). Dexmedetomidine protects against apoptosis induced by hypoxia/reoxygenation through the inhibition of gap junctions in NRK-52E cells. Life Sci. 122, 72–77. 10.1016/j.lfs.2014.12.009 25529146

[B24] Marettova E. Maretta M. Legath J. Kosutzka E. (2012). The retention of cadmium and selenium influence in fowl and chickens of F1 generation. Biol. Trace Elem. Res. 147 (1-3), 130–134. 10.1007/s12011-011-9305-5 22201045

[B25] Martins-Marques T. Catarino S. Zuzarte M. Marques C. Matafome P. Pereira P. (2015). Ischaemia-induced autophagy leads to degradation of gap junction protein connexin43 in cardiomyocytes. Biochem. J. 467 (2), 231–245. 10.1042/bj20141370 25605500

[B26] Martins-Marques T. Pereira P. Girao H. (2014). P145 Degradation of gap junction protein Cx43 by autophagy in ischemic heart is determined by the triggering signal: the role of AMPK vs Beclin1. Cardiovasc. Res. 103 (Suppl. l), S25 10.1093/cvr/cvu082.84

[B27] Meng S. F. Mao W. P. Wang F. Liu X. Q. Shao L. L. (2015). The relationship between Cd-induced autophagy and lysosomal activation in WRL-68 cells. J. Appl. Toxicol. 35 (11), 1398–1405. 10.1002/jat.3114 25639782

[B28] Merrifield C. J. Kaksonen M. (2014). Endocytic accessory factors and regulation of clathrin-mediated endocytosis. Cold Spring Harb. Perspect. Biol. 6 (11), a016733 10.1101/cshperspect.a016733 25280766 PMC4413230

[B29] Nordberg G. F. (2009). Historical perspectives on cadmium toxicology. Toxicol. Appl. Pharmacol. 238 (3), 192–200. 10.1016/j.taap.2009.03.015 19341754

[B30] Rikans L. E. Yamano T. (2000). Mechanisms of cadmium-mediated acute hepatotoxicity. J. Biochem. Mol. Toxicol. 14 (2), 110–117. 10.1002/(SICI)1099-0461(2000)14:2<110::AID-JBT7>3.0.CO;2-J 10630425

[B31] Shi T. Song W. Xu R. (2017). Autophagy and ER stress in LPS/GalN-induced acute liver injury. Mol. Med. Rep. 16 (5), 7001–7005. 10.3892/mmr.2017.7409 28901440

[B32] Solan J. L. Lampe P. D. (2018). Spatio-temporal regulation of connexin43 phosphorylation and gap junction dynamics. Biochim. Biophys. Acta Biomembr. 1860 (1), 83–90. 10.1016/j.bbamem.2017.04.008 28414037 PMC5640473

[B33] Song X.-B. Liu G. Wang Z.-Y. Wang L. (2016). Puerarin protects against cadmium-induced proximal tubular cell apoptosis by restoring mitochondrial function. Chem. Biol. Interact. 260, 219–231. 10.1016/j.cbi.2016.10.006 27717697

[B34] Sun L. Gao J. Zhao M. Cui J. Li Y. Yang X. (2015). A novel cognitive impairment mechanism that astrocytic p-connexin 43 promotes neuronic autophagy via activation of P2X7R and down-regulation of GLT-1 expression in the hippocampus following traumatic brain injury in rats. Behav. Brain Res. 291, 315–324. 10.1016/j.bbr.2015.05.049 26031379

[B35] Tang H. Da L. Mao Y. Li Y. Li D. Xu Z. (2009). Hepatitis B virus X protein sensitizes cells to starvation-induced autophagy via up-regulation of beclin 1 expression. Hepatology 49 (1), 60–71. 10.1002/hep.22581 19065679

[B36] Vinken M. Doktorova T. Decrock E. Leybaert L. Vanhaecke T. Rogiers V. (2009). Gap junctional intercellular communication as a target for liver toxicity and carcinogenicity. Crit. Rev. Biochem. Mol. Biol. 44 (4), 201–222. 10.1080/10409230903061215 19635038

[B37] Vivian S. Lau A. F. (2016). Connexins: mechanisms regulating protein levels and intercellular communication. FEBS Lett. 588 (8), 1212–1220 *.* 10.1016/j.febslet.2014.01.013 PMC3989427 24457202

[B38] Wang J. Zhu H. Zhang C. Wang H. Yang Z. (2018). Baicalein ameliorates cadmium-induced hepatic and renal oxidative damage in rats. Indian J. Anim. Res. 53 (4), 523–527. 10.18805/ijar.B-853

[B39] Wang L. Y. Fan R. F. Yang D. B. Zhang D. Wang L. (2019). Puerarin reverses cadmium-induced lysosomal dysfunction in primary rat proximal tubular cells via inhibiting Nrf2 pathway. Biochem. Pharmacol. 162, 132–141. 10.1016/j.bcp.2018.10.016 30347204

[B40] Wang X.-Y. Yang H. Wang M.-G. Yang D.-B. Wang Z.-Y. Wang L. (2017). Trehalose protects against cadmium-induced cytotoxicity in primary rat proximal tubular cells via inhibiting apoptosis and restoring autophagic flux. Cell Death Dis. 8 (10), e3099 10.1038/cddis.2017.475 29022917 PMC5682644

[B41] Wang X. Zhang J. Fu J. Wang J. Ye S. Liu W. (2015). Role of ROS-mediated autophagy in radiation-induced bystander effect of hepatoma cells. Int. J. Radiat. Biol. 91 (5), 452–458. 10.3109/09553002.2015.1012308 25651038

[B42] Xing J. Z. Zhu L. J. Jackson J. A. Gabos S. Sun X. J. Wang X. B. (2005). Dynamic monitoring of cytotoxicity on microelectronic sensors. Chem. Res. Toxicol. 18 (2), 154–161. 10.1021/tx049721s 15720119

[B43] Zhang C. Lin J. Ge J. Wang L.-L. Li N. Sun X. T. (2017). Selenium triggers Nrf2-mediated protection against cadmium-induced chicken hepatocyte autophagy and apoptosis. Toxicol in Vitro 44, 349–356. 10.1016/j.tiv.2017.07.027 28765097

[B44] Zou H. Liu X. Han T. Hu D. Wang Y. Yuan Y. (2015a). Salidroside protects against cadmium-induced hepatotoxicity in rats via GJIC and MAPK pathways. PLoS One 10 (6), e0129788 10.1371/journal.pone.0129788 26070151 PMC4466396

[B45] Zou H. Liu X. Han T. Hu D. Yuan Y. Gu J. (2015b). Alpha-lipoic acid protects against cadmium-induced hepatotoxicity via calcium signalling and gap junctional intercellular communication in rat hepatocytes. J. Toxicol. Sci. 40 (4), 469 10.2131/jts.40.469 26165643

[B46] Zou H. Zhuo L. Han T. Hu D. Yang X. Wang Y. (2015c). Autophagy and gap junctional intercellular communication inhibition are involved in cadmium-induced apoptosis in rat liver cells. Biochem. Biophys. Res. Commun. 459 (4), 713–719. 10.1016/j.bbrc.2015.03.027 25778869

[B47] Zou H. Wang T. Yuan J. Sun J. Yuan Y. Gu J. (2020). Cadmium-induced cytotoxicity in mouse liver cells is associated with the disruption of autophagic flux via inhibiting the fusion of autophagosomes and lysosomes. Toxicol. Lett. 321, 32–43. 10.1016/j.toxlet.2019.12.019 31862506

